# The role of inflammatory proteins in regulating the impact of lipid specifications on deep venous thrombosis: a two sample and mediated Mendelian randomization study

**DOI:** 10.3389/fcvm.2024.1434600

**Published:** 2024-08-20

**Authors:** Fan Dong, Jiahao Sun, Yudong Zhang

**Affiliations:** ^1^ The First Clinical Medical College of Shandong University of Traditional Chinese Medicine, Jinan, Shandong, China; ^2^ Jiangsu Province Hospital of Chinese Medicine, Affiliated Hospital of Nanjing University of Chinese Medicine, Nanjing, Jiangsu, China; ^3^ Department of Vascular Surgery, Affiliated Hospital of Shandong University of Traditional Chinese Medicine, Jinan, Shandong, China

**Keywords:** lipid species, inflammatory proteins, deep vein thrombosis, Mendelian randomization, mediation role

## Abstract

**Objective:**

To investigate the potential mediating role of inflammatory proteins in the association between lipid species and Deep Venous Thrombosis (DVT).

**Methods:**

A comprehensive analysis was conducted using pooled data from genome-wide association studies (GWAS), incorporating double-sample and reverse Mendelian randomization (MR) techniques, to identify the specific inflammatory proteins that act as intermediaries among 91 screened proteins in relation to deep vein thrombosis (DVT). Furthermore, a two-step MR approach was employed to quantify the proportion of DVT risk attributed to lipid effects mediated by these inflammatory proteins.

**Results:**

The MR Analysis revealed that the two inflammatory proteins, as predicted by genetics, served as mediating factors in the impact of five lipids on DVT. No reverse effect of DVT was observed on 179 lipid species and 91 inflammatory proteins. In the case of TAG(58:7) and its influence on DVT, CCL20 played an intermediary role with an estimated proportion of 12.51% (ranging from 12% to 13%). SIRT2 exhibited a masking effect on DVT for PC(17:0/20:4) and PC(18:0/20:4), while CCL20 masked the impact of DVT on PC(14:0/18:2), PC(15:0/18:2), and PC(18:0/20:5).

**Conclusions:**

In our study, we identified CCL20 as a crucial mediator in the association between TAG(58:7) and DVT, with a mediating proportion of 12.51% (12%-13%). Further investigations are warranted to explore other potential risk factors acting as mediators.

## 1 Introduction

Deep vein thrombosis (DVT) is a vascular disorder characterized by abnormal blood clotting within the deep veins, resulting in lumen obstruction and impaired venous blood return. Among various clinical manifestations, DVT predominantly affects the lower extremities, with a relatively high incidence observed following vascular surgery. Perturbations in lipid metabolism and inflammatory factors significantly contribute to the pathogenesis and progression of DVT (1–3).

Kawasaki T (4) postulated that hyperlipidemia and hypercholesterolemia are established risk factors for deep vein thrombosis (DVT). Commonly employed lipid metabolism indicators encompass Total Cholesterol (TC), Triglyceride (TG), high density lipoprotein cholesterol (HDL-C), low density lipoprotein cholesterol (LDL-C), very low density lipoprotein cholesterol (VLDL-C), apolipoprotein A-1 (apoA-1), and lipoprotein(a) [LP(a)]. Owing to the intricate diversity in lipid structure and function, lipidomics has emerged as a distinct field. According to LIPIDMAPS classification, lipids can be further categorized into eight groups, namely fatty acyls, glycerolipids, sterol lipids, prenol lipids, sphingolipids, glycolipids, saccharolipids, and polyketides. Integrating metabolomics with lipidomics can also offer valuable insights into small-molecule signatures of disease etiology particularly in individuals predisposed to atherosclerosis where there exists a strong intra-class correlation between lipid metabolites and amino acid metabolites (5).

Inflammation is the physiological response of the host to infection or injury. Numerous studies have consistently demonstrated that thrombosis is accompanied by an inflammatory reaction, wherein inflammatory factors exert their effects on vascular endothelial cells, leading to endothelial damage and initiating thrombus formation. Therefore, inflammation is intricately linked with the occurrence and progression of deep vein thrombosis (DVT). The orchestration of the inflammatory response involves a complex network comprising various cells and mediators, including circulating proteins such as cytokines and soluble receptors. In a study conducted by J.E.P et al. (6)91 protein quantitative trait loci (pQTL) associated with inflammation were identified, and these findings were integrated with disease genome-wide association studies (GWASs) to elucidate the functional consequences of disease-associated genetic variants. By employing Mendelian randomization (MR) analysis and co-localization techniques, causally implicated proteins in immune-mediated diseases etiology were discerned.

This study employed Mendelian randomization analysis to further investigate the association between lipidomics, inflammatory factor-related proteins, and deep vein thrombosis (DVT), elucidating the potential role of inflammatory factor proteins in mediating the impact of lipid types on DVT pathogenesis. These findings offer novel insights for clinical prevention and treatment strategies targeting DVT.

## 2 Data and methods

### 2.1 Flowchart

**Figure 1:**
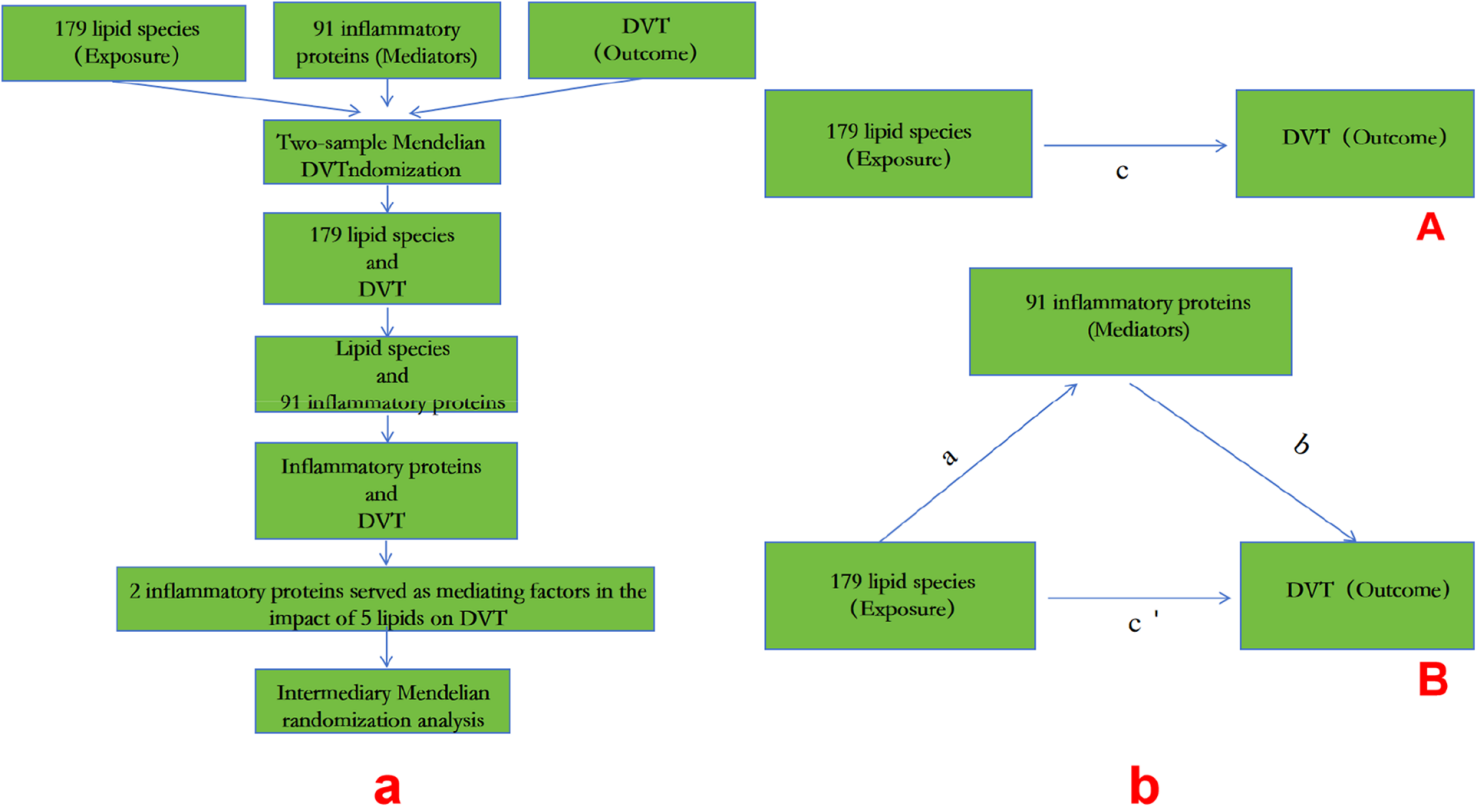
A: MR process; b: **(A)** the total effect between lipid species and DVT.c is the total effect using genetically predicted lipid species as exposure and DVT as outcome. d is the total effect using genetically predicted DVT asexposure and lipid species as outcome. **(B)** The total effect was decomposed into: (i) indirect effect using a two-step approach (where a is the total effect of lipid species on inflammatory proteins, and b is the effect of inflammatory proteins on DVT)and the product method (a × b) and (ii) direct effect (c′ = c – a × b). Proportion mediated wasthe indirect effect divided by the total effect.

The flowchart and intermediary Mendelian randomization analysis are shown in Figure 1.

### 2.2 Data

The GWAS datasets of 179 lipid species, 91 inflammatory proteins, and DVT were utilized as exposures in the two-sample, reverse, and MR studies, as elaborated in Supplementary Table S1.

This study constituted a secondary data review of publicly available databases with appropriate ethical approval and informed consent obtained. The writing process adhered to the requirements outlined by STROBE-MR, the reporting code for MR studies; detailed information regarding the data can be found in Supplementary Table S1.

### 2.3 Mendelian randomization analysis

The successful implementation of a Mendelian randomization study relies on the fulfillment of three crucial assumptions: (1) Hypothesis 1: The selected single nucleotide polymorphism should exhibit a significant correlation with the exposure variable. (2) Hypothesis 2: Single nucleotide polymorphisms must be independent of potential confounding factors that may exist between the exposure and outcome variables. (3) Hypothesis 3: Single nucleotide polymorphisms are not directly associated with the outcome variable, but rather can only establish a causal link through their influence on the exposure factors (7, 8).

Firstly, the single nucleotide polymorphism (SNP) sites extracted from the genome-wide association study (GWAS) data of 179 lipid species, 91 inflammatory proteins, and deep vein thrombosis (DVT) exhibit genome-wide significance with a threshold of *P* < 5 × 10–8. To avoid bias, linkage imbalance is eliminated through careful consideration. Furthermore, linkage disequilibrium analysis is conducted using a threshold of r2 = 0.001 and kb = 5 000 to ensure independence of the instrumental variables. Additionally, confounding between variables is effectively controlled for, ensuring that exposure does not introduce additional confounding effects and that there are no interactions between exposure and mediator variables (9).

According to the hypothesis of Mendelian randomization analysis, instrumental variable single nucleotide polymorphisms should exhibit a strong association with exposure. To assess the strength of instrumental variables, we calculated the F statistic for each individual single nucleotide polymorphism. A value of F > 10 indicates minimal bias from weak instrumental variables (10). The formula for calculating F is as follows: F = [(n - K - 1)]/K × [r2/(1 - r2)], where N represents the sample size of GWAS exposed, K is the number of single nucleotide polymorphisms, r2 denotes the proportion of variation explained by single nucleotide polymorphisms in the exposure database, and r2 = 2 × (1-MAF) × MAF × *β*/SD. Herein, MAF refers to minor allele frequency (equivalent to EAF), *β* represents the effect value of the allele (*β*), and SD = sx×√N where sx denotes standard error.

In this Mendelian randomization analysis, the inverse variance weighted (IVW) method is employed as the primary approach, which aggregates genotype-based data. The weighted median estimator (WME) and MR-Egger methods are utilized to enhance the accuracy of IVW estimates, assess the consistency of Mendelian randomization results, and identify and correct for horizontal heterogeneity. WME allows for inclusion of potentially invalid single nucleotide polymorphisms (SNPs), provided that their instrumental variable strength exceeds 50%. The SNP weights are ranked, with the median value serving as the final estimate. MR-Egger regression accommodates pleiotropy in all SNPs and can detect horizontal heterogeneity through intercept testing while providing adjusted estimates after correcting for pleiotropy. Sensitivity analysis employs Cochran's Q test to evaluate heterogeneity in individual genetic variation estimates. Leave-one-out sensitivity tests calculate Mendelian randomization results by excluding one instrumental variable at a time. If there is substantial discrepancy between these results and the overall findings estimated using other instrumental variables, it indicates sensitivity of the Mendelian randomization results to that particular instrumental variable (11, 12).

Two independent samples and reverse Mendelian randomization were employed to identify the causal factors underlying deep vein thrombosis (DVT) in relation to 179 lipid species and 91 inflammatory proteins. Subsequently, a screening was conducted to identify the inflammatory proteins associated with the lipid species. Causality was assessed using various methods including inverse variance weighted method, weighted median method (WM), simple median method (SM), weighted median estimator (WME), and MR-Egger regression. Odds ratios were calculated and presented in a forest plot (13, 14).

Furthermore, mediating Mendelian randomization analysis was performed to determine the extent of mediation by the 91 inflammatory proteins on the effect of the 179 lipid species on DVT. Additionally, multivariate MR analysis was conducted to assess the independent effects of screened inflammatory proteins and lipid species on DVT by incorporating all exposures into a single model. Significant correlated SNPs were identified and combined with existing exposure variables for further analyses after excluding duplicate SNPs. The causal effects of each SNP along with their corresponding standard errors were estimated using IVW methods based on weighted linear regression in multivariate MR analyses.

### 2.4 Statistical analysis

All statistical analyses were performed using R version 4.2.3, MR 0.7.0 and TwoSample MR 0.5.6 software packages. MR analysis was performed and statistical plots were drawn. The results were expressed by odds ratio (OR) and 95%con reference interval (95% CI). *P* < 0.05 was considered as significant difference. In the sensitivity analysis, Cochran's Q (*P* > 0.05) meant that no significant heterogeneity was observed in the study, and MR-Egger intercept (*P* > 0.05) meant that there was no gene pleiotropy in the study, thus satisfying the core hypothesis of MR.

## 3 Results

### 3.1 Relationship between 179 lipid species and DVT

**Figure 2:**
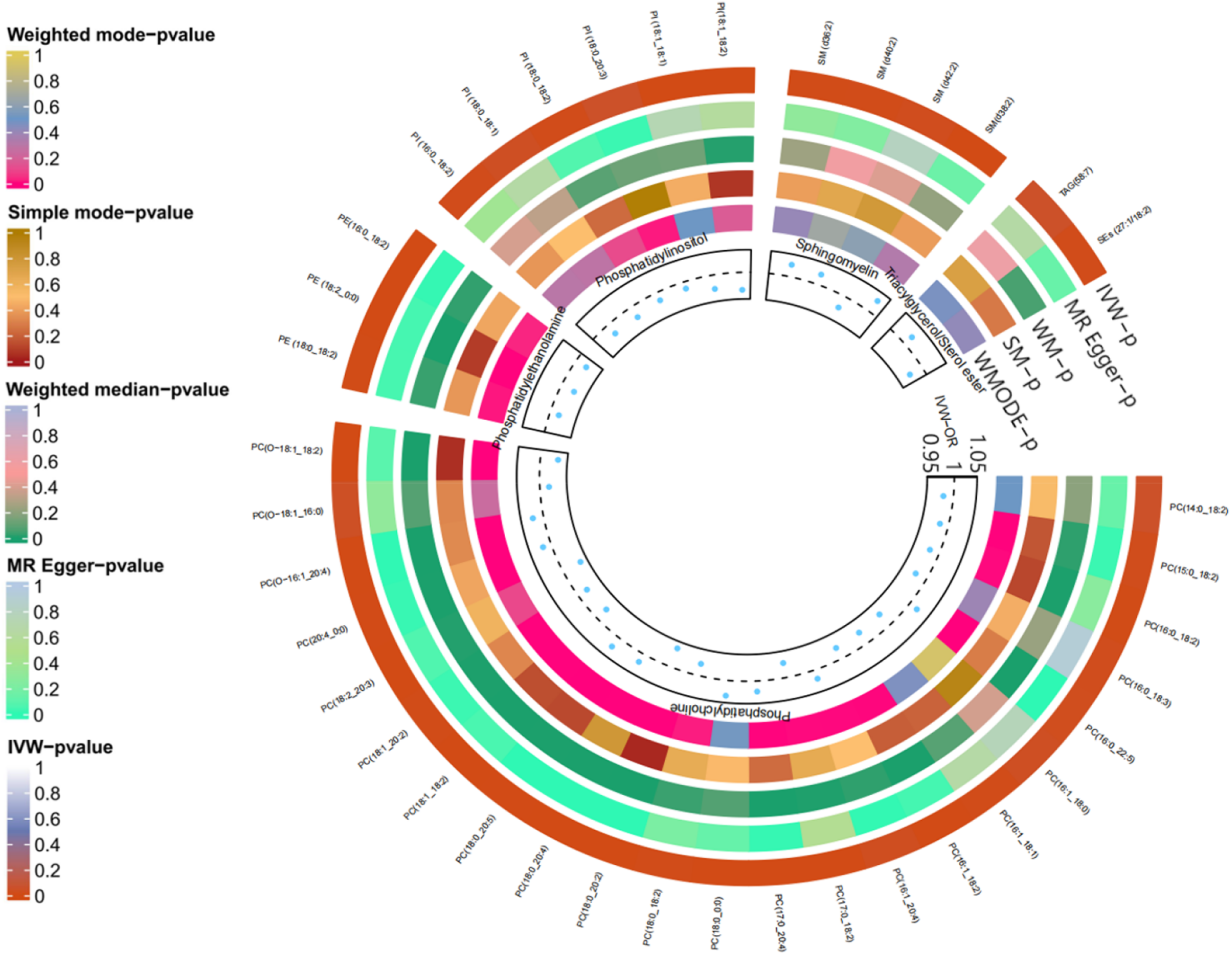
Two sample MR forest plot.

As anticipated, we observed significant genetic associations with DVT for 38 out of 179 lipid species (*P* < 0.05). Specifically, there were 23 phosphatidylcholine (PC), 6 phosphatidylinositol (PI), 4 sphingomyelin (SM), and 3 phosphatidylethanolamine (PE) species identified as associated with DVT risk. Additionally, one sterol ester (SEs) and one triacylglycerol (TAG) exhibited significant associations, while the remaining lipid species were excluded from further analysis; please refer to Figure 2 for detailed information.

### 3.2 Relationship between 91 inflammatory proteins and DVT

**Figure 3:**
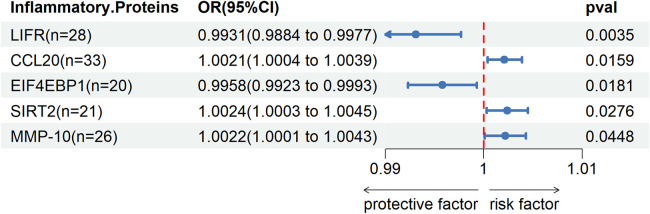
Two sample MR IVW forest plot: relationship between 91 inflammatory proteins and DVT.

Through two-sample MR Cycling, we identified a significant genetic association (*P* < 0.05) between five inflammatory proteins and DVT. Among these proteins, Leukemia inhibitory factor receptor (LIFR) and Eukaryotic translation initiation factor 4E-binding protein 1 (EIF4EBP1) were found to exert a protective effect against DVT, while C-C motif chemokine 20 (CCL20), SIR2-like protein 2 (SIRT2), and Matrix metalloproteinase-10 (MMP-10) were identified as risk factors for DVT.Please refer to Figure 3 for detailed information.

### 3.3 Relationship between 38 lipid species and 5 inflammatory proteins

**Figure 4:**
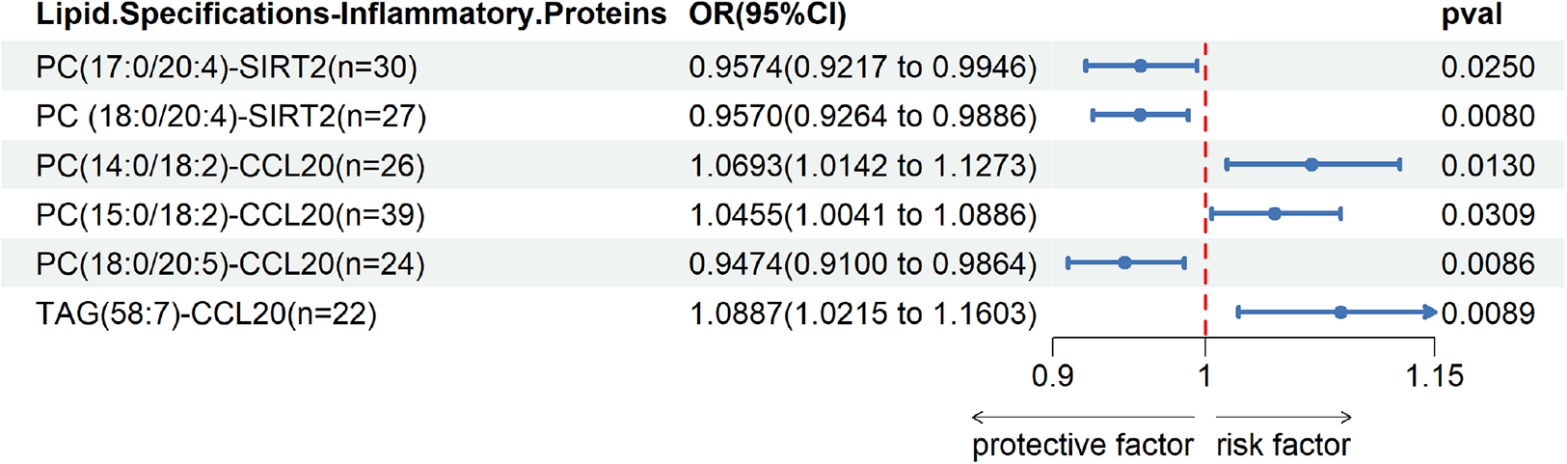
Two sample MR IVW forest plot: relationship between 38 lipid species and 5 inflammatory proteins.

We observed cycling between 38 lipid species and 5 inflammatory proteins in two samples. Significant correlations were found between PC(17:0/20:4) and SIRT2, PC(18:0/20:4) and SIRT2, PC(14:0/18:2) and CCL20, PC(15:0/18:2) and CCL20, PC(18:0/20:5) and CCL20, as well as TAG(58:7) and CCL20 Union; the remaining associations were not statistically significant.Please refer to Figure 4 for detailed information.

### 3.4 Reverse Mendelian randomization results

There was no strong evidence that genetically predicted DVT had an effect on 179 lipid species and 91 inflammatory proteins (*P* > 0.05).

### 3.5 Mediating Mendelian randomization results

After excluding the influencing factors, we took PC(17:0/20:4) and SIRT2, PC(18:0/20:4) and SIRT2, PC(15:0/18:2) and CCL20, PC(18:0/20:5) and CCL20, TAG(58:7) and CCL20 for mediation analysis. Table 1 show the effect of SIRT2 mediated PC(17:0/20:4), PC(18:0/20:4) and CCL20 mediated PC(14:0/18:2), PC(15:0/18:2), PC(18:0/20:5) and TAG(58:7) on DVT in the mediation analysis. It is noteworthy that the c “value is opposite to the direct effect (C-C”) in the SIRT2-mediated effect of PC(17:0/20:4) and PC(18:0/20:4) on DVT, and CCL20-mediated effect of PC(14:0/18:2), PC(15:0/18:2) and PC(18:0/20:5) on DVT On the contrary. Therefore, SIRT2 plays a masking role in the relationship between 179 lipid species and DVT. CCL20 played a mediating role in the effect of TAG(58:7) on DVT, accounting for 12.51%(12%-13%). Please refer to Table 1 for detailed information.

**Table 1:** Intermedisary MR results.

Exposure	Mediators	Outcome	c	a	b	c'		Mediated proportion
			Effect size	Effect size	Effect size	Effect size	SE	(%) (95%CI)
PC(17:0/20:4)	SIRT2	DVT	0.0015	−0.0435	0.0024	−0.0001	0.0008	7.17% (6.99%-7.33%)
PC (18:0/20:4)			0.0013	−0.0440	0.0024	−0.0001	0.007	8.29% (8.14%-8.42%)
PC(14:0/18:2)	CCL20		−0.0014	0.0670	0.0021	0.0001	0.0018	10% (9.65%-10.37%)
PC(15:0/18:2)			−0.0012	0.0445	0.0021	0.0001	0.0009	8.21% (8.09%-8.33%)
PC(18:0/20:5)			0.0016	−0.054	0.0021	−0.0001	0.0011	7.03% (6.83%-7.02%)
TAG(58:7)			0.0014	0.0850	0.0021	0.0001	0.0028	12.51% (12%-13%)

### 3.6 Multivariate Mendelian randomization results

The multivariate MR analysis was performed by integrating 6 lipid species and 2 inflammatory proteins. The results revealed that, after accounting for confounding effects and excluding the impact of repeated SNP, there was no significant gene correlation between the six lipid species, two inflammatory proteins, and DVT (*P* < 0.05).

## 4 Discussion

The pathogenesis of DVT is multifactorial, with recent research focusing on the interplay between lipid species, inflammatory proteins, and DVT. Abnormal lipid metabolism is a recognized risk factor for DVT formation, while thrombosis represents an inflammatory reaction process. Inflammatory factors and coagulation factors collaborate to form a dense network that promotes disease occurrence and progression (10, 15). Although clinical studies have observed associations between blood lipids, inflammatory factors, and DVT, these results may be confounded by various factors. Leveraging existing GWAS, we employed MR analysis to investigate the causal relationship between lipid species and DVT mediated through inflammatory proteins.

Firstly, our findings from two samples and reverse Mendelian randomization (MR) analysis demonstrated significant correlations between 38 lipids and 5 inflammation-related proteins predicted by genes with the risk of deep vein thrombosis (DVT) (*P* < 0.05). Secondly, MR analysis of these 38 lipids and 5 inflammation-related proteins revealed intercorrelations among 6 lipid species and 2 inflammatory proteins. Finally, we conducted intermediary MR analysis using selected GWAS data, which identified CCL20 as an intermediary factor in the association between TAG(58:7) and DVT risk, accounting for approximately 12.51% (12%–13%) of the total effect.

TAG is a lipid component in the blood, which can interfere with the occurrence of DVT through the following aspects. Increased blood viscosity: High levels of triacylglycerol cause increased blood viscosity, which increases the risk of thrombosis; Endothelial dysfunction: Hypertriglyceridemia (high triglyceride levels) is associated with endothelial dysfunction. When endothelial cells are damaged or dysfunctional, they may not properly regulate blood coagulation and platelet activation, thereby promoting the formation of DVT. Proinflammatory effects: High levels of triacylglycerol are associated with an inflammatory response. Triglyceride may aggravate the risk of DVT by promoting inflammatory response. Promotion of oxidative stress: Hypertriglyceridemia is also associated with increased oxidative stress. Oxidative stress can promote the activation of platelet and coagulation system. Other metabolic abnormalities: High triglyceride levels are usually associated with other metabolic abnormalities, such as obesity, hypertension, diabetes, etc., which are themselves risk factors for DVT (16, 17).

The protein C-C motif chemokine 20 (CCL20) belongs to the CC chemokine family and exerts diverse functions, particularly within the immune system. CCL20 levels critically influence immune cell migration and activation, thereby significantly impacting inflammatory responses, infectious diseases, and oncogenesis. Elevated CCL20 concentrations can attract a greater number of immune cells to sites of inflammation, exacerbating the inflammatory response. Moreover, in certain malignancies, heightened CCL20 expression may be associated with tumor invasion and metastasis (18, 19).

The occurrence of DVT is often associated with vascular wall damage, reduced blood flow velocity, and a hypercoagulable state of the blood. Abnormally elevated levels of TAG(58:7) in the human body may impact the expression and function of CCL20 through various pathways. TAG(58:7) can potentially induce increased production of CCL20 by activating specific signaling pathways or regulating gene expression. As a chemotactic factor, CCL20 has the ability to attract and activate immune cells such as monocytes and lymphocytes, thereby recruiting them to the site of injury. These cells play a crucial role in initiating an inflammatory response, which can further exacerbate inflammation within the vascular wall leading to endothelial injury and an increased risk of thrombosis formation. Excessive levels of CCL20 may trigger an exaggerated inflammatory response and even contribute to autoimmune disease development. Additionally, CCL20 could also influence blood coagulation status and promote thrombus formation.

Secondly, SIRT2 in PC(17:0/20:4), PC(18:0/20:4) and CCL20 in PC(14:0/18:2), PC(15:0/18:2), PC(18:0/20:5) play a masked role in DVT. Firstly, according to the direction of the total effect, they were divided into two groups. Firstly, in the effect of CCL20 on PC(14:0/18:2) and PC(15:0/18:2) on DVT, PC(14:0/18:2) and PC(15:0/18:2) on DVT were protective effects. That is, the increase of PC(14:0/18:2) and PC(15:0/18:2) levels will reduce the risk of DVT. However, the increase of PC(14:0/18:2) and PC(15:0/18:2) levels will lead to the increase of CCL20 level (OR > 1), and the increase of CCL20 level will further lead to the increase of DVT risk (OR > 1). Secondly, the role of SIRT2 in PC(17:0/20:4), PC(18:0/20:4) and CCL20 in PC(18:0/20:5) on DVT. The increase of PC(17:0/20:4), PC(18:0/20:4) and PC(18:0/20:5) levels will increase the risk of DVT. However, the increase of PC(17:0/20:4) and PC(18:0/20:4) will lead to the decrease of SIRT2 level, and the increase of PC(18:0/20:5) will lead to the decrease of CCL20 level (0 < OR < 1), and the decrease of SIRT2 and CCL20 levels will lead to the decrease of DVT risk. This situation is not uncommon in medicine, as molecules and signaling pathways in organisms are often intertwined and influence each other. A molecule may affect a disease or pathological process through multiple pathways, and sometimes the effects of these pathways may even be opposite. Our study presents, for the first time, evidence of a masking effect between SIRT2 and CCL20, shedding new light on their interaction in cell signaling and disease processes. As a deacetylase, SIRT2 plays a crucial role in regulating cell cycle and metabolic balance. On the other hand, as a chemokine, CCL20 is involved in inflammation and immune response. We hypothesize that this masking effect may be influenced by intracellular signaling pathways or transcription factor activity; however, the specific molecular mechanism requires further exploration. Future investigations should focus on elucidating the direct intracellular interactions between SIRT2 and CCL20 and how they mutually influence each other's functions through shared signaling pathways or regulatory networks. Additionally, it is essential to validate the involvement of these molecules in specific disease mechanisms while exploring their potential as therapeutic targets.Secondly, there are different dimensions of DVT, that is, different causes of DVT, and SIRT2 and CCL20 have different mutation genes. Will this have different effects on DVT? This may partly explain the masking effect of SIRT2 and CCL20. Whether there are other confounding factors affecting the results of MR Still needs further study.

It is worth noting that multivariate MR analysis revealed no significant genetic associations between the 6 lipid species and 2 inflammatory proteins with DVT. Furthermore, Mendelian randomization analysis demonstrated a small effect size for these lipid species and inflammatory proteins on DVT, suggesting their impact may not be particularly substantial. However, this does not imply their effects on DVT are negligible. The reason being that the lipidomics and inflammation-related proteins we investigated are more refined and comprehensive than traditional blood substances and inflammatory factors. These substances, such as PC, PI, SM, etc., likely participate in physiological processes including blood coagulation, vascular function, and inflammatory response—all of which are closely linked to DVT development. Changes in these factors may therefore influence the risk of developing DVT. Nevertheless, it should be noted that individual factors alone may have limited significance due to the multifactorial nature of DVT formation. Moreover, human physiology is highly complex; even minor changes can trigger cascading reactions. The odds ratio (OR) value merely indicates the strength of association between a factor and DVT rather than its probability of causing DVT directly. Henceforth, despite having small OR values, the potential impact of these factors on DVT cannot be completely disregarded or deemed unimportant; further investigation is warranted to gain a more comprehensive understanding of their role in the pathogenesis of DVT.

## 5 Limitation

Our study has several limitations. Firstly, the MR Approach offers a robust tool for inferring causality; however, its translation into clinical practice is limited. The intricate and long-term cumulative impact of genetic variations poses challenges in directly guiding immediate clinical interventions. Moreover, the applicability of GWAS results to specific patient populations may be uncertain due to small effects from individual genetic mutations. In order to advance precision medicine and align research methods with clinical needs, interdisciplinary collaboration is imperative for further exploring the intricate relationship between genetics and diseases.Secondly, despite our efforts to identify and eliminate confounding variables, we cannot completely exclude the possibility that horizontal pleiotropy influenced our results. Thirdly, in this study, we utilized publicly available aggregate data from a European population database. However, the use of aggregated data instead of individual-level data may introduce certain limitations. The overall dataset might not fully capture the variations and specific characteristics among individuals, potentially leading to biases and errors during data collection and processing. Consequently, due to the inability to comprehensively reflect individual differences and specificities, our study results possess a certain degree of generalizability but lack specificity. This restricts the extrapolation of our conclusions and highlights the limitations associated with the dataset used. To address these limitations and enhance the generalizability of our findings, future studies should employ more diverse sampling strategies that encompass participants from various geographic regions as well as different ethnic and racial backgrounds. By doing so, not only can we improve result representation but also gain insights into how genetic and environmental disparities across populations influence specific phenotypes or disease risks.Fourthly, due to using aggregate level statistics instead of individual level data in this study, we were unable to further investigate causality between subgroups such as women and men. Additionally, inflammatory proteins played a masking role in our mediation analysis with a relatively small proportion of mediators; therefore, further research is warranted to comprehend underlying reasons for these findings. Finally, it should be acknowledged that we did not differentiate between different sites and causes of DVT in detail; however, determinants of DVT may vary according to specific sites and causes which could potentially impact our results.

## 6 Conclusion

In conclusion, this study utilizes genetic data in MR Analysis to provide compelling evidence. Based on the analysis of two samples and reverse MR, we have identified 38 lipid specifications and 5 inflammatory proteins that are genetically associated with DVT. Among the effects of TAG(58:7) on DVT, CCL20 acts as a crucial intermediary with an intermediary ratio of 12.51% (ranging from 12% to 13%). Additionally, SIRT2 masks the association between DVT and PC(17:0/20:4) as well as PC(18:0/20:4), while CCL20 masks the association between DVT and PC(14:0/18:2), PC(15:0/18:2), and PC(18:0/20:5).

## Data Availability

The original contributions presented in the study are included in the article/Supplementary Material, further inquiries can be directed to the corresponding author.
