# Supplementary material for: The role of inflammatory proteins in regulating the impact of lipid specifications on deep venous thrombosis: a two sample and mediated Mendelian randomization study
Source: Front Cardiovasc Med. 2024 Aug 20;11:1434600. doi: 10.3389/fcvm.2024.1434600 (PMC11369674; doi:10.3389/fcvm.2024.1434600)
Supplement: Supplementary file 1 [file Table1.docx]

Supplementary Table 1:Data sources

| Exposure/Outcome/Mediating factor | Sample size | Sample  ethnic origin | PubMed ID or URL | Data publication year |
| --- | --- | --- | --- | --- |
| 179 lipid species | 377,277 | European | PMID:37907536 | 2023 |
| 91inflammatory proteins | 14824 | European | PMID:37563310 | 2023 |
| DVT | 484,598 | European | PMID:33959723 | 2021 |
